# Pharmacokinetic Comparison of Three Different Administration Routes for Topotecan Hydrochloride in Rats

**DOI:** 10.3390/ph13090231

**Published:** 2020-09-02

**Authors:** Seung-Hyun Jeong, Ji-Hun Jang, Yong-Bok Lee

**Affiliations:** College of Pharmacy, Chonnam National University, 77 Yongbong-ro, Buk-gu, Gwangju 61186, Korea; rhdqn95@naver.com (S.-H.J.); jangji0121@naver.com (J.-H.J.)

**Keywords:** topotecan hydrochloride, administration routes, pharmacokinetic studies, non-clinical study, comparison

## Abstract

Topotecan is actively used in clinic, with its primary use being in treatment of various types of cancer. The approved administration routes are oral and intravenous. The purpose of this study was to investigate and identify pharmacokinetic profiles of different administration routes. We conducted pharmacokinetic studies on three different routes of administration in rats. Five rats in each group received a single dose of 4 mg/kg of topotecan hydrochloride intravenously, orally, or subcutaneously, and the concentrations of lactone and total forms of the drug in plasma, urine, and feces were quantified. Various pharmacokinetic parameters were compared statistically. Plasma concentrations of both the lactone and total forms at elimination phase following subcutaneous administration, were two times higher than was seen with oral administration and 10 times higher than with intravenous administration. Subcutaneous administration of topotecan showed pharmacokinetic profiles similar to sustained release. In addition, subcutaneous administration showed bioavailability from 88.05% (for lactone form) to 99.75% (for total form), and these values were four–five times greater than those of oral administration. The results of this non-clinical study will not only provide greater understanding of the in vivo pharmacokinetics of topotecan, but also be useful for development of additional formulations and/or administration routes.

## 1. Introduction

Topotecan is an antitumor agent that belongs to a chemical family called plant alkaloids [1]. Cancer cells are characterized by abnormal cell division, and unlike normal cells, they lose their control of cell division and are out of balance [2]. In general, cancer cells are killed by damaging the ribonucleic acid (RNA) or deoxyribonucleic acid (DNA) necessary for cell division so that they can no longer divide [3]. Topotecan is a cell cycle selective anticancer agent that inhibits cell division by preventing DNA synthesis, through inhibition of topoisomerase [4]. This drug is mainly used for ovarian cancer, cervical cancer, and lung cancer. It can also be used for other tumors [5,6].

Topotecan is capable of reversible hydrolysis of lactone ring sites within its structure depending on the surrounding pH [7]. As pH is lowered, the lactone form with a closed ring prevails. On the contrary, as pH increases, the carboxylated form of topotecan with the ring open, prevails. Interestingly, the lactone and the carboxylated forms differ in their pharmacological activity, and the anticancer activity of the lactone form is greater than that of the carboxylated form [8]. The chemical structures of both forms are shown in Figure S1.

For the purpose of chemotherapy, topotecan is administered orally as a capsule or intravenously in combination with fluids in the clinic [9]. Improving the bioavailability of topotecan would have both economic and therapeutic benefits. In general, the price of anticancer drugs is burdensome to patients and the maintenance of effective concentrations for treatment in chemotherapy is very important. In order to solve these problems, one approach is to develop new drugs and to improve existing formulations, but it may also be possible to search for new and better routes of administration of existing drugs. In addition, studying new routes for administration will help to better understand the pharmacokinetic properties of drugs, and based on this, improved formulations and new drug development may be possible. If the concentration over time values are the same for different routes of administration, it may be advantageous from the therapeutic perspective for topotecan to persist in the blood rather than to present large amounts intermittently. In other words, for cell cycle selective inhibition, it may be important to maintain continuous high blood concentrations of the drug. Maintaining drug concentrations in the blood may lead to continuous inhibition of topoisomerase, preventing DNA synthesis in rapidly dividing cancer cells. Thus (although more research will be needed in the future, including clinical trials), topotecan may be a drug that requires the development of subcutaneous or controlled release formulations that are long-lasting in the blood.

We focused on these points in this study. The pharmacokinetics of other routes in addition to topotecan’s existing routes was investigated and the results were compared to focus on the potential for new routes with advantageous pharmacokinetics.

Very few pharmacokinetic studies of additional routes of topotecan administration (except oral and intravenous) have been reported. We explored additional routes of administration in addition to existing routes, and focused on subcutaneous injections as a result. Intravenous administration has the advantage of very high bioavailability (due to no exceptional absorption barrier and process), but also has the disadvantages of increasing the risk of systemic side effects and invasive processes [10]. In addition, the use of an intravenous catheter requires the intervention of a skilled medical professional and the pain and fear that patients feel can be significant [11]. Oral administration has the advantages of being very simple, usually with excellent compliance, but has the disadvantage of relatively low bioavailability due to first pass effect in liver and drug degradation in gastrointestinal tract [12]. Subcutaneous administration, by contrast, has some potential to neutralize the advantages and disadvantages of intravenous and oral administration [13]. It is less invasive and has lower systemic side effects than intravenous administration, and bioavailability may be significantly improved compared to oral administration. Uckun et al. administered topotecan hydrochloride to severe combined immuno-deficiency mice subcutaneously for 72 h using a micro-osmotic pump, and presented a plasma concentration profile in a steady state [14]. However, in the study of Uckun et al., access to pharmacokinetic information other than the mean plasma concentration and distribution volume in steady state was difficult.

There have been no detailed reports on topotecan pharmacokinetics that included excretion patterns (through feces and urine) in addition to blood concentration profiles by routes of administration. In addition, no pharmacokinetics have been reported for the topotecan lactone form and other forms by routes of administration. As mentioned above, the topotecan is either closed (as lactone form) or opened (as carboxylated form) with lactone rings in the structure, depending on the pH environment in vivo. These processes are reversible and the lactone form has better activity than the carboxylated form. Therefore, it is important to analyze the active and relatively inactive forms (of topotecan) in biological samples. In this study, we quantified not only the lactone form of topotecan but also the total topotecan (converted from the carboxylated form to the lactone form) from various biological samples. This may help to answer questions about the abundance (of the active form) and conversion ratio (from active form to non-active form) of topotecan in biological samples. In addition to reversible hydrolysis by pH, topotecan has been reported to have liver metabolism by CYP enzymes [9,15]. Metabolic pathways include N-dealkylation (producing N-demethyltopotecan) and glucuronidation. However, hepatic metabolism of topotecan, mediated by CYP isoenzymes, is of minor quantitative importance. That is, only small amounts of topotecan are demethylated (or glycosylated) in the liver [9,15]. Therefore, this study focused on pharmacokinetic research by quantifying lactone and carboxylated forms as the main in vivo metabolism of topotecan.

The purpose of this study was to identify and investigate (in terms of pharmacokinetics of both lactone and total form of topotecan) potential routes of administration by identifying the pharmacokinetic profiles of different routes of administration of topotecan hydrochloride. In a significant sense, our current non-clinical experiments (including in vivo excretion) are expected to be of great help in the development of better formulations and therapies for topotecan in the future. Anticancer drugs are most often administered by intravenous injection, but for some, clinical application has been delivered by other routes of administration including oral, subcutaneous, intramuscular, intraperitoneal, and intrathecal [16]. In particular, anti-cancer drugs that are delivered subcutaneously include cytarabine [17], bortezomib [18], azacitidine [19], and rituximab [20], which can also be administered intravenously. As mentioned above, oral and intravenous administration of topotecan has been approved for clinical use. However, there have been few clear studies or reports on the efficacy of subcutaneous administration of topotecan. Moreover, we have not found any clear reports that administering topotecan subcutaneously has significant side effects or is contraindicated in clinical practice. Pescosolido et al. reported significant changes in the shape and location of retinal vessels after intravenous and subcutaneous administration of topotecan hydrochloride (1 mg/kg dose) to retinoblastoma model rats [21]. That is, as a result of subcutaneous and intravenous administration of topotecan to retinoblastoma model rats, the antitumor effect was suggested through anti-angiogenesis of retinal vascular. Furthermore, Pescosolido et al. reported no mortality and minor side effects in both subcutaneous and intravenous groups despite of the administration (of topotecan) to newborn rats [21].

## 2. Results

The ultraperformance liquid chromatography-electrospray ionization-tandem mass spectrometer (UPLC-ESI-MS/MS) method was preferentially established in this study to quantitatively analyze the lactone or total form of topotecan from biological samples (plasma, urine, feces). The UPLC-ESI-MS/MS assay used in this study was established with reference to previously reported topotecan bioassays [22,23] with some modifications and improvements. In particular, mobile phase composition, gradient elution program, run time, and extraction solvent composition were optimized for our analysis system. Moreover, the assay was extended to quantify topotecan from urine and fecal samples as well as plasma. The UPLC-ESI-MS/MS method established in this study was validated according to FDA bioassay guidelines [24]. This method analyzes topotecan at a run time of 4 min per sample (a relatively short analysis time), enabling efficient pharmacokinetic studies on topotecan.

The precursor and product ion mass spectra of topotecan and repaglinide (as an internal standard, IS) were derived from a scan mode of the individual standard solution into a mass spectrometer. Topotecan and repaglinide generated protonated molecular ions as [M + H]^+^ in positive-ion mode. The most abundant (with consistent and stable) fragment ion for multiple reaction monitoring (MRM) was *m*/*z* 422.10 → 377.15 for topotecan lactone form and *m*/*z* 453.20 → 230.30 for IS. This MRM transition of topotecan lactone form was consistent (as 422.1 → 377 in positive MRM mode) with previous reports [22,23]. Figure S2 shows the relevant mass spectra (including selected precursor and product ions). The results related to the selectivity (or specificity) of the assay are presented in Figure S3. As shown in Figure S3, there were no major interferences with endogenous substances around the retention time of the analytes in the blank matrices. The linearities of calibration curves for topotecan were excellent (as *r*^2^ > 0.99) in rat plasma, urine, and feces (ranging from 0.1 to 1000 ng/mL). Figure S4 shows calibration curves in rat plasma, urine, and feces. In addition, the lower limit of quantitation (LLOQ) for topotecan was very low (0.1 ng/mL in rat plasma, urine, and feces), and was sufficient for pharmacokinetic studies after administration (through three different administration routes) of topotecan to rats. Intra-batch accuracies for topotecan ranged from 97.00% to 101.87% with a precision (coefficient of variation; CV) of <6.66%. Inter-batch accuracies for topotecan ranged from 97.67% to 102.40% with a precision (CV) of <6.86%. Table S1 shows the accuracy and precision results of topotecan quantification in intra- and inter-batch. The recoveries of topotecan from biological samples (rat plasma, urine, and feces) were 88.90–92.00%. The matrix effects of topotecan from biological samples (rat plasma, urine, and feces) were 96.10–100.32%. Table S2 shows the recovery and matrix effect results in topotecan assay. The stabilities under various conditions of topotecan from biological samples (rat plasma, urine, and feces) were 96.33–101.05%. Table S3 shows the stability results of topotecan in rat matrices. The stock solutions for topotecan and IS were stable in the storage concentration for 4 weeks at −20 °C. The stabilities of topotecan and IS stock solutions were 98.89% ± 2.15% and 99.94% ± 1.90%, respectively. As a result, the accuracy and precision of the method and the recovery, matrix effect, and stabilities of the topotecan were all within acceptable limits. We also determined the dilution integrity for topotecan, and the sample dilution did not affect the accuracy and precision (within ± 15%) of topotecan analysis.

A total of 15 Sprague-Dawley male rats were used in the study, which were randomly divided into three groups (intravenous, oral, and subcutaneous administration). All groups had a single dose of topotecan hydrochloride dissolved in sterile distilled water at the same dose (4 mg/kg), with an injection volume of 1 mL/kg. All groups had the same sampling time points for blood, urine, and feces. No specific (visually distinguished) adverse effects (including local damage and redness at the site of administration) were observed in any of the rats after administration of topotecan hydrochloride intravenously, orally, or subcutaneously.

**Figure 1:**
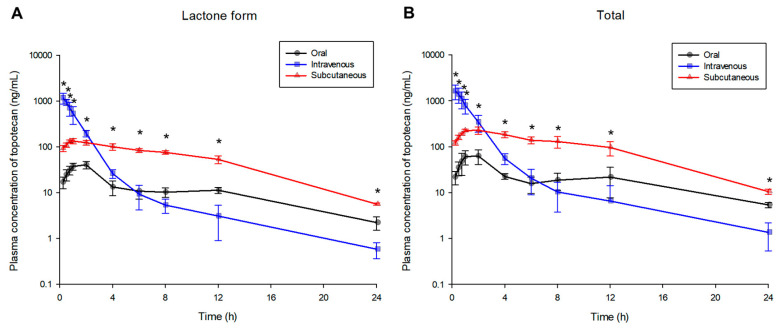
Mean plasma concentration-time profiles of topotecan (as lactone form (**A**) and total topotecan (**B**)) after oral (-●-, black color), intravenous (-■-, blue color), and subcutaneous (-▲-, red color) administration of 4 mg/kg topotecan hydrochloride to rats (*n* = 5). Vertical bars represent standard deviation of the mean. * *p* < 0.05 (by ANOVA).

The plasma concentration-time profiles (as lactone form and total topotecan) observed after intravenous, oral, and subcutaneous administration of topotecan hydrochloride are shown in Figure 1. In the case of subcutaneous administration, the plasma concentrations of (both lactone and total form) topotecan were significantly higher (*p* < 0.05; in post-hoc analysis) than those of oral administration at all time points from 0 to 24 h (including absorption, distribution, and elimination phases) after administration. In addition, in the case of subcutaneous administration, the plasma concentrations of (both lactone and total form) topotecan were significantly higher (*p* < 0.05; in post-hoc analysis) than those of intravenous administration from 4 to 24 h (including elimination phase) after administration. Topotecan (both lactone and total form) plasma concentrations of subcutaneous administration from 4 to 24 h after administration were significantly higher (*p* < 0.05; in post-hoc analysis) than oral administration and much higher than intravenous administration (especially from 8 to 24 h). Topotecan given subcutaneously showed a pharmacokinetic profile similar to that of delayed (or sustained) release. The patterns of plasma pharmacokinetic profiles of the topotecan lactone form and the total topotecan were almost similar and only differed in the concentration values (total topotecan concentrations were higher than the lactone form).

The average area under the plasma concentration-time curve (AUC) from 0 to 24 h after administration (AUC_0–24_) values (for lactone form topotecan) obtained after oral, intravenous, and subcutaneous administration of 4 mg/kg of topotecan hydrochloride were 283.71, 1629.02, and 1404.53 h·ng/mL, respectively. The mean maximum plasma concentration (C_max_) values (for lactone form topotecan) obtained after oral, intravenous, and subcutaneous administration were 42.62, 1167.54, and 142.36 ng/mL, respectively. The mean time to reach C_max_ (T_max_) values (for lactone form topotecan) obtained after oral and subcutaneous administration were 1.60 and 1.35 h, respectively. In this regard, the pharmacokinetic parameter values (including urinary and fecal excretion study results) of topotecan (as lactone form) are presented in Table 1 while total topotecan values are presented in Table 2. The average AUC_0–24_ values (for total topotecan) obtained after oral, intravenous, and subcutaneous administration were 537.87, 2530.59, and 2465.78 h·ng/mL, respectively. The mean C_max_ values (for total topotecan) obtained after oral, intravenous, and subcutaneous administration were 69.79, 1632.14, and 246.07 ng/mL, respectively. The mean T_max_ values (for total topotecan) obtained after oral and subcutaneous administration were 2.00 and 1.40 h, respectively.

**Figure 2:**
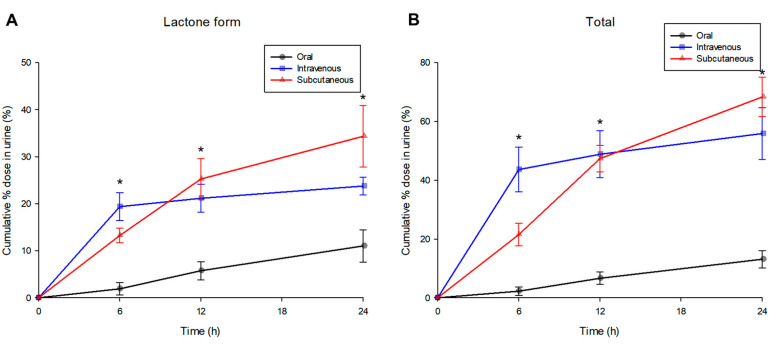
Mean (% dose) cumulative urinary excreted amount-time profiles of topotecan (as lactone form (**A**) and total topotecan (**B**)) after oral (-●-, black color), intravenous (-■-, blue color), and subcutaneous (-▲-, red color) administration of 4 mg/kg topotecan hydrochloride to rats (*n* = 5). Vertical bars represent standard deviation of the mean. * *p* < 0.05 (by ANOVA).

The cumulative (as % dose) urinary excreted amounts-time profiles of topotecan (as lactone form and total topotecan) observed after intravenous, oral, and subcutaneous administration are shown in Figure 2. A significant amount of topotecan administered into the body was excreted via urine. Topotecan (both lactone and total form) excretion through urine up to 24 h after administration was significantly higher in subcutaneous administration than oral and intravenous administration (*p* < 0.05; in post-hoc analysis). For intravenous administration, a significant amount of (both lactone and total form) topotecan was excreted (to urine) within 6 h after administration. For subcutaneous and oral administration, a significant amount of (both lactone and total form) topotecan was excreted with urine up to 12 h after administration (however, excretion continued until 24 h after administration). Topotecan urinary excretion profiles of subcutaneous and oral administration were similar (although there were differences in excretion amounts), but somewhat different from intravenous administration.

**Figure 3:**
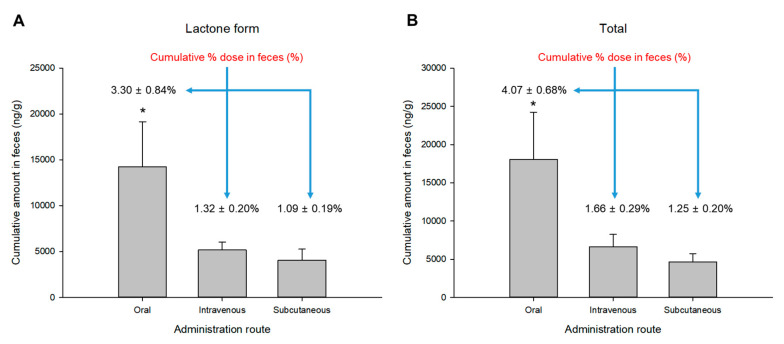
Mean cumulative fecal excreted amount (at 24 h after administration) of topotecan (as lactone form (**A**) and total topotecan (**B**)) after oral, intravenous, and subcutaneous administration of 4 mg/kg topotecan hydrochloride to rats (*n* = 5). Vertical bars represent standard deviation of the mean. * *p* < 0.05 (by ANOVA).

The cumulative fecal amounts (up to 24 h after administration) of topotecan (as lactone form and total topotecan) observed after intravenous, oral, and subcutaneous administration of topotecan hydrochloride are shown in Figure 3. The excretion of (both lactone and total form) topotecan into feces (up to 24 h after administration) was significantly higher (*p* < 0.05; in post-hoc analysis) in oral administration, and subcutaneous administration (as mean excretion value) was less than with intravenous administration (as mean excretion value). The urinary and fecal excretion profiles of the topotecan lactone form and the total topotecan were very similar and only differed in the excretion values (total topotecan excretion amounts were higher than the lactone form).

## 3. Discussion

Because these experiments required analyzing a large number of biosamples, a simpler sample preparation method was preferred, and was applied based on modifications to the sample preparation used in [22]. Protein precipitation using methanol showed relatively good recovery of topotecan from biosamples and was very simple and fast. Quantification of total topotecan was relatively easy and simple through acidification of biosamples using 10% (*v*/*v*) aqueous formic acid. In previous studies, the same method was used to efficiently quantify total topotecan from plasma [22,23]. In this study, there was little difference when comparing the topotecan peak area in the acidified sample with the same concentration of the topotecan standard solution. On the other hand, the peak area of the topotecan in the samples without acidification was smaller than in the standard solution of the same concentration of topotecan. These results indicated that the reversible conversion efficiency of topotecan from carboxylated form to lactone form through acidification of biosamples was significant. Validation of the established UPLC-ESI-MS/MS method was performed primarily with quality control samples in four concentration intervals (LLOQ, low, medium, and high), and confirmed recovery, stability, and matrix-effect as well as inter-batch and intra-batch accuracy and precision. In addition, the linearity of the calibration curves was also confirmed. In this study, repaglinide was used as the IS. This is because repaglinide showed excellent peak-shaped symmetry and recovery under the set analysis conditions (including mobile phase, column, and sample preparation). And above all, the retention time of repaglinide (as 2.36 min) did not overlap (which may cause interference between mutual reactions such as cross-talk) with retention time of topotecan (as 2.09 min). In addition, structurally, repaglinide and topotecan have similarities in that they contain amine groups, hydroxyl groups, and aromatic rings in their structures and have similar molecular weights (shown in Figure S2), and as a result, largely heterogeneous parts in chromatographic and physicochemical properties (including extraction and ionization) were not identified.

The AUC_0–24_/AUC_0–∞_ × 100 values were 89.79–99.59% in both the topotecan lactone form and the total topotecan lactone form (shown in Table 1 and Table 2). Therefore, the sensitivity and blood sampling of the assay used in this study were adequate.

Subcutaneous administration of topotecan did not differ significantly in AUC compared to intravenous administration and maintained (relatively) high blood levels for a significant time following administration. Lactone and total topotecan plasma concentrations of subcutaneous administration at elimination phase after administration were two times higher than oral administration and 10 times higher than intravenous administration (Table 1 and Table 2). The pharmacokinetic profile obtained after subcutaneous administration of topotecan hydrochloride was similar to a delayed (or sustained) release of the drug. In other words, after subcutaneous administration of topotecan hydrochloride, the AUC and C_max_ (of both lactone and total form of topotecan) were higher (*p* < 0.05; in post-hoc analysis), and T_max_ was reached faster, compared with oral administration. AUC values (of both lactone and total form of topotecan) calculated after subcutaneous administration of topotecan hydrochloride were similar to those seen with intravenous administration (*p* > 0.05; in post-hoc analysis). The plasma concentrations from 4 to 24 h (including C_24 h_ values) determined after subcutaneous administration of topotecan hydrochloride were significantly higher (*p* < 0.05; in post-hoc analysis) than those of intravenous and oral administration. In addition, topotecan (both forms) bioavailability (F, %) after subcutaneous administration was four–five times higher than that of oral administration. These results are probably due to the advantages of the route of administration. In other words, subcutaneous administration is slower in the blood absorption of the drug than intravenous administration, but the drug can be absorbed relatively quickly into blood vessels developed around the subcutaneous tissue. Subcutaneous administration acts as a reservoir of drugs, allowing for sustained absorption and release. Moreover, above all, subcutaneous administration can prevent drug metabolism (or hydrolysis) in the gastrointestinal tract and avoid the first pass effect in the liver compared to oral administration. Since there are generally more metabolic enzymes in the gastrointestinal tract than in subcutaneous tissues [25], and topotecan is reported to be metabolized mainly in the liver [26,27], oral administration will be more susceptible to metabolism and degradation of topotecan.

The higher values of C_max_ and AUC transform ratios (as (C_max_ of total − C_max_ of lactone form)/C_max_ of total × 100 and/or (AUC_0–24_ of total − AUC_0–24_ of lactone form)/AUC_0–24_ of total × 100) in oral and subcutaneous administration compared to intravenous administration may be related to metabolism and/or hydrolysis from the lactone form of the topotecan to the carboxylate form in other tissues (such as gastrointestinal tract for oral administration and subcutaneous tissues for subcutaneous administration). The transform ratio values of AUC for subcutaneous administration were lower than for oral administration (shown in Table 2). Although not so great, the difference in the transform ratio between subcutaneous and oral administration will be related to the first pass effect and intestinal metabolism following oral administration, as mentioned above. In the intravenous route, the transform ratio of topotecan was lower than that of oral and subcutaneous, but the ratio by metabolism such as demethylation may be higher (in the intravenous administration). In this regard, further studies on metabolites such as demethylation of topotecan will be needed.

For the lactone form, an average of 11.02–34.33% of the dose administered orally, intravenously, or subcutaneously was excreted via urine. For total topotecan, an average of 13.10–68.31% of the dose administered orally, intravenously, or subcutaneously was excreted through urine. Overall, a significant amount of topotecan administered into the body was excreted through urine. In particular, subcutaneous administration of topotecan hydrochloride revealed more excretion (of both lactone and total form of topotecan) than oral and intravenous (*p* < 0.05; in post-hoc analysis). These differences in the excretion of each group are also thought to be due to the characteristics of the administration route. In other words, subcutaneous administration of topotecan hydrochloride maintained higher concentrations (of topotecan) in the blood than intravenous and oral, which may have resulted in more urinary excretion (through the kidney). In addition, as shown in Figure 1, intravenous administration showed higher topotecan concentrations in plasma until 2 h after administration, at which point they fell to the lowest plasma concentration among the administration routes from 6–8 h after administration. These results likely relate to the excretion profile of topotecan through urine. That is, excretion of topotecan through urine up to 6 h after administration was the largest in intravenous administration and tended to be larger in subcutaneous administration from 12–24 h after administration. Furthermore, in the case of intravenous administration, the excretion of topotecan was mostly done at 0–6 h after administration, but subcutaneous and oral administration continued until 0–24 h after administration. As a result, it is thought that there is a correlation between the plasma concentration profile and urine excretion profile (of both forms).

In contrast, fecal excretion was less than urine, and among the three administration routes, it was highest with oral administration and lowest with subcutaneous administration. However, there was no significant difference in excretion to feces between subcutaneous and intravenous administration. There was also no significant difference in the mean % (of administered dose) value of topotecan excretion to feces under subcutaneous and intravenous administration. These differences in the excretion of each group are also thought to be due to the characteristics of the administration route. More orally administered topotecan hydrochloride was excreted through the gastrointestinal tract to feces than subcutaneous and intravenous delivery. In addition, when administered intravenously and subcutaneously to rats, the quantification of topotecan in feces (as shown in Figure 3) suggested the enterohepatic circulation of topotecan. Enterohepatic circulation has been reported in mice in the past [28]. There has also been a report of enterohepatic circulation of topotecan in a human pharmacokinetic study [29].

In the elimination phase of the plasma concentration-time profile obtained after oral administration of topotecan hydrochloride, rebound of topotecan into the blood was observed (as shown in Figure 1). Further research on the exact mechanism of rebound may be required, but this may also be closely related to the topotecan’s enterohepatic circulation.

The total recovery of topotecan to urine and feces by oral administration was less than 25% (up to 24 h after dose). On the other hand, the total recoveries of topotecan to urine and feces following intravenous and subcutaneous administration were approximately 60% (up to 24 h after dose). Calculating (cumulative % dose in urine × bioavailability) the excretion % dose in urine after oral administration based on intravenous administration was approximately 13%, which was consistent with the experimental results (as 13.10% ± 2.97%) of this study. Fecal excretion should be approximately 0.4% (as % dose) when calculated in the same way (cumulative % dose in feces × bioavailability) as in urine, but the experimental result was 4.07% ± 0.68%. It is believed that fecal excretion increased due to enterohepatic circulation after oral administration. This was also consistent with the observed rebound plasma concentration in oral administration in Figure 1. Therefore, although the degree is not so great, it is thought that the topotecan undergoes enterohepatic circulation.

Results similar to ours have been reported for other drugs. According to Lau et al., midazolam was administered subcutaneously, intraperitoneally, and orally to rats, and the highest AUC and bioavailability (39.3%) values were found in subcutaneous administration [30]. Thus, Lau et al. concluded that subcutaneous administration is an alternative route, not only because it yielded the greatest bioavailability, but also for its reliability and safe use [30]. When ivermectin was administered orally and subcutaneously to sheep and horses, higher blood levels were maintained (for a long duration after drug administration) with subcutaneous administration, and in terms of efficacy, better results (antiparasitic effects) were obtained [31]. Methotrexate showed about 1.5 times higher bioavailability (in rheumatoid arthritis patients) when administered subcutaneously than orally, and it was confirmed that higher blood concentration was maintained with subcutaneous administration. They concluded that clinical routes of parenteral (including subcutaneous route) administration of methotrexate needs to be considered [32]. In the case of moxidectin, the AUC values of subcutaneous and oral administration were similar, but the efficacy (as anthelmintic effects) of subcutaneous administration was better than that of oral administration, and the mean residence time of the drug was significantly longer [33]. Other studies have reported that intravenous and subcutaneous administration of bortezomib to relapsed multiple myeloma patients has shown that subcutaneous administration offers non-inferior efficacy to standard intravenous administration, with an improved safety (such as low occurrence of peripheral neurotoxicity) profile [18,34]. In addition, Leveque et al.’s review report suggested that subcutaneous administration should be considered as an alternative parenteral route for anticancer drugs [35]. Additionally, according to Leveque et al., subcutaneous injection as a route of administration of anticancer drugs has many advantages including a treatment effect similar to intravenous administration, fewer side effects, ambulatory treatment, and self-administration [35].

In our non-clinical studies here, significant pharmacokinetic differences among orally, intravenously, and subcutaneously administered topotecan hydrochloride in rats were clarified. In the future, safety and efficacy assessments (especially in humans) of topotecan’s subcutaneous route of administration will need to be conducted. As clinical side effects of topotecan, neutropenia, thrombocytopenia, anemia, leucopenia, anorexia, alopecia, fatigue, weakness, malaise, pruritus, pyrexia, hyperbilirubinemia, and rash have been reported. Therefore, subcutaneous administration of topotecan may cause side effects such as skin redness and hypersensitivity, which are equivalent to or higher than oral and intravenous administration. However, as mentioned above, there have been no reports of fatal toxicity following subcutaneous administration of topotecan, and there have been some non-clinical studies of subcutaneous administration of topotecan [14,21]. Furthermore, the results of these non-clinical studies have not identified serious side effects in experimental animals (even though topotecan was injected subcutaneously continuously for 72 h). In addition, no serious adverse effects were observed in our study, including topical damage and redness at the site of subcutaneous administration of topotecan hydrochloride to rats. Considering the improved pharmacokinetic profiles of subcutaneous administration (compared to oral and intravenous administration) identified in this study, it may be possible to reduce the degree of side effects through various methods such as lowering the dose of topotecan or adjusting the administration cycle. That is, when the topotecan is administered subcutaneously, the same effect may be maintained even at a small dose compared to the oral and intravenous doses. Conversely, if topotecan should be administered orally and intravenously daily, it may be administered subcutaneously every other day to maintain the same efficacy as oral and intravenous administration.

Future research will need to expand on the development of a physiologically based pharmacokinetic (PBPK) model (that includes subcutaneous routes of administration in addition to oral and intravenous) for topotecan. Moreover, in the developed PBPK model, dosage calculation and therapeutic prediction (of topotecan) in humans will be possible through interspecific extrapolation and reverse dosimetry.

## 4. Materials and Methods

### 4.1. Materials and Reagents

Reference standards for topotecan hydrochloride (purity ≥ 99%) and repaglinide (purity ≥ 99%) as IS were purchased from Sigma-Aldrich (St. Louis, MO, USA). Heparin sodium (25,000 IU/5 mL) was bought from Choongwae Pharm. Co. (Seoul, Republic of Korea). LC-MS grade methanol, acetonitrile, and water (18.2 MΩ) were purchased from Fisher Scientific (Hampton, NH, USA). LC-MS grade formic acid was obtained from Sigma-Aldrich (St Louis, MO, USA). All chemicals had the highest LC-MS grade or quality available.

### 4.2. Analytical Methods

Plasma, urine, and feces concentrations of topotecan were determined using an established UPLC-ESI-MS/MS method. The analytical methods used in this study (including chromatographic conditions and sample preparation) were optimized and applied to our analytical conditions by referring to previous studies [22,23] that most closely matched our system. Briefly, 10 μL of IS (repaglinide, 10 ng/mL in plasma) and 500 μL of cold (−20 °C) methanol were added to 100 μL of samples (plasma, urine, and feces). Here, in the case of feces, distilled water corresponding to five times (mL/g) the total feces weight (g) was added and crushed by a homogenizer. Thereafter, only the supernatant was taken by centrifugation (10,000× *g* for 10 min). Urine samples were also diluted five-fold (*v*/*v*) with blank urine (obtained from six or more individuals).

After being vortex-mixed for 5 min, the samples were centrifuged at 13,500× g for 5 min. Then, 500 μL of the supernatant organic layer was dried gently with a centrifugal vacuum evaporator under nitrogen gas at 40 °C for 3 h. The dried matter was reconstituted with 50 μL of mobile phase solution and vortexed for 5 min. Finally, to quantify the topotecan lactone form, after centrifugation at 13,500× *g* for 5 min, 5 μL of the supernatant was injected into the UPLC-ESI-MS/MS system. In addition, the biological samples obtained were acidified with 10% (*v*/*v*) aqueous formic acid to quantify the total topotecan (including the conversion from carboxylated form to lactone form). The subsequent sample preparation was the same as above.

UPLC-ESI-MS/MS consisted of a Shimadzu Nexera-X2 Series UPLC system (Shimadzu, Kyoto, Japan) coupled with a Shimadzu-8040 mass spectrometer with a DGU-20A degassing unit and an SIL-30AC autosampler. Optimized chromatographic separation of topotecan was conducted with a Kinetex Core-Shell C_18_ (50 × 2.1 mm i.d. (inner diameter), 1.7 μm particle size; Phenomenex Inc., Torrance, CA, USA) column at an oven temperature of 40 °C. The mobile phase consisted of 0.1% formic acid in water (mobile phase A) and acetonitrile (mobile phase B). Analysis was performed with a gradient elution and a flow rate of 0.3 mL/min. The elution program was as follows: 0–0.75 min (10% B), 0.75–1.50 min (10–90% B), 1.50–2.50 min (90% B), 2.50–2.51 min (90–10% B), and 2.51–4.0 min (10% B). All analytical procedures were evaluated with positive electrospray ionization (ESI). And quantification was achieved using MRM modes at *m*/*z* 422.10 → 377.15 for topotecan (as lactone form) and *m*/*z* 453.20 → 230.30 for IS. Acquisition and analysis of data were achieved using a LabSolutions program (Shimadzu, Kyoto, Japan) with collision energy of −20 and −27 eV for topotecan and IS, respectively. The run time per sample was 4 min.

### 4.3. Validation of Analytical Methods

The selectivity of the assay was confirmed by the responses of peaks derived after analysis of blank (samples containing no analytes), zero (samples including only IS), LLOQ (samples containing IS and LLOQ concentration topotecan), and obtained samples after administration of topotecan.

The calibration curves were prepared separately using the corresponding blank matrices for each matrix (as plasma, urine, and feces). The blank matrices here were from six or more rats. Calibration curves were prepared using the eight calibration points by linear regression with weighting factor of 1/concentration^2^. The linearity was calculated by plotting the analyte/IS peak area versus the theoretical concentration of analyte. A linear calibration equation with its correlation coefficient (*r*^2^) was generated.

Intra-batch accuracy and precision were measured by analyzing the quality control (QC) samples at five different times on the same day (*n* = 5). Inter-batch assessments were similarly carried out on five consecutive days (*n* = 5). The QC samples here were prepared separately using the corresponding blank matrices as the calibration curve samples. The concentration of each QC sample was evaluated using freshly prepared calibration standards. The precision was found by determining the CV for the analysis of the QC samples. The CV of precision for each concentration level was not allowed to deviate by more than ± 15% except for the LLOQ, with a limit of 20%. The accuracy was examined based on the following criteria: the mean value not exceeding 15% of the nominal concentration except for the LLOQ, which had to be not greater than 20%.

The matrix effect and recovery of topotecan were examined for the QC samples at low, medium, and high concentrations in five replicates (*n* = 5). Here, low, medium, and high were 0.3, 500, and 800 ng/mL (as topotecan), respectively. The extraction recovery was determined by comparing the peak area obtained from extracted blank rat matrices spiked with QCs with that of samples spiked after extraction at corresponding concentrations. The recovery did not need to be 100%, but the value should have been reproducible, consistent, and precise. The matrix effect was determined by comparing the peak area of samples spiked after extraction with that of the absolute standard solution containing the equivalent amount of topotecan. A matrix effect value of 100% meant that the matrix components had little effect on the analysis of topotecan.

The stabilities of topotecan in rat biological samples were examined under various conditions, including short-term, long-term, freeze-thaw, and autosampler (post-preparative) stability. Two different levels of QC samples, low (0.3 ng/mL for topotecan) and high (800 ng/mL for topotecan) concentrations, were analyzed for all stability tests. The long-term stability was obtained by analysis of QC samples frozen for 4 weeks at −80 °C, and the short-term stability test was conducted by maintaining the QC samples for 24 h at room temperature (25 °C). For the freeze and thaw stability test, the QC samples were stored for 24 h at −80 °C and then thawed completely at 25 °C. This cycle was repeated, and the analysis was performed after third cycle. For the post-preparative stability test, the QC samples were placed in the autosampler for 24 h at 15 °C. The stabilities of the stock solutions of topotecan and IS were evaluated by determining the concentrations of analytes after being stored for 4 weeks at −20 °C. Samples were considered stable if the mean peak area value at each level was within ± 15% of the sample nominal concentration, and the precision was less than 15% (*n* = 5).

The dilution integrity test was conducted to confirm that dilution by adding the same biological matrices when the concentration of topotecan in the sample exceeded the upper limit of quantification (>1000 ng/mL) did not affect the analysis. We had demonstrated dilution integrity by the standard analyte (for topotecan) with concentrations above the upper limit of the standard curve, spiked with blank matrices. We measured dilution integrity in five replicates (*n* = 5).

### 4.4. Pharmacokinetic Studies

We performed topotecan pharmacokinetic studies on three routes of administration (intravenous, oral, and subcutaneous) for this study. Animal experiments were approved by Chonnam National University Animal Experimental Ethics Committee, Republic of Korea (approval number: CNU IACUC-YB-2017-44). This study was performed according to the revised Guidelines for Ethical Conduct in the Care and Use of Animals and the rules of Good Laboratory Practice. Five Sprague-Dawley male rats (8 weeks, 240–260 g) in each group received a single dose of 4 mg/kg of topotecan hydrochloride intravenously, orally, and subcutaneously, and plasma, urine, and fecal samples were obtained at planned times. Intravenous administration was performed to the lateral tail vein and oral administration was performed by rat zonde (needle for oral administration). Subcutaneous administration was performed at the nape of the rat (using a 1 mL syringe). All rats were housed separately in metabolic cages in a ventilated animal room with controlled temperature (23 ± 1 °C) and relative humidity (50% ± 5%). They were kept on a 12 h/12 h light/dark cycle and provided free access to food and water. All rats were fasted overnight before drug administration. However, the rats had free access to water. During that time, blank urine and feces samples were collected. Blood samples of approximately 0.2–0.25 mL were collected at the jugular vein of free alive rat before administration (0 h) and at 0.25, 0.5, 0.75, 1, 2, 4, 6, 8, 12, and 24 h after administration (including oral, intravenous, and subcutaneous). During the blood sampling period, all rats were free to drink water. Collected blood samples were placed into microtubes (Axygen, Inc., Union, CA, USA) containing 5 μL of heparin solution to prevent blood clotting. Plasma was immediately separated by centrifuging blood at 10,000× g for 10 min and stored at −80 °C until further analysis. To clarify the excretion of topotecan via feces and urine, urine samples were collected at intervals of 0–6, 6–12, and 12–24 h and feces were collected at 24 h after drug administration. The volume and weight of urine samples and feces were accurately measured and recorded and stored at −80 °C until analysis.

C_max_ and T_max_ were individually determined using plasma concentration-time curve. AUC_0-24_ was calculated by the linear trapezoidal rule. The AUC from 0 to infinity (AUC_0–∞_) was calculated as AUC_0–24_ + C_24 h_/k, where C_24 h_ was the last measurable concentration (in this study) and k was the elimination rate constant at terminal phase. The half-life (T_1/2_) was calculated as 0.693/k. The systemic clearance (CL) and volume of distribution (V_d_) were calculated as dose/ AUC_0–∞, intravenous_ and dose/k·AUC_0–∞, intravenous_, respectively. Absolute oral bioavailability (F, %) was calculated as AUC_0–∞, oral_/AUC_0–∞, intravenous_ × 100. In addition, absolute subcutaneous bioavailability (F, %) was calculated as AUC_0–∞, subcutaneous_/AUC_0–∞, intravenous_ × 100. All pharmacokinetic parameters were calculated by noncompartmental analysis using WinNonlin^®^ software (version 8.1, Pharsight^®^, a Certara™ Company, San Diego, CA, USA). The cumulative amounts of topotecan excreted in feces up to 24 h were expressed as total excreted amounts and a percentage of the topotecan hydrochloride administered dose. The cumulative amounts of topotecan excreted in urine up to 24 h were expressed as total excreted amounts and a percentage of the topotecan hydrochloride administered dose. All data were expressed as mean ± standard deviation (SD). The above pharmacokinetic parameter values were calculated (or determined) for each form of topotecan (lactone and total).

### 4.5. Statistical Analysis Method

Statistical analysis was performed using the Statistical Package for the Social Sciences (SPSS) software version 23 (IBM, Armonk, NY, USA) on the pharmacokinetic parameters obtained from different administration routes. In other words, all pharmacokinetic parameters (including plasma concentrations and excretion amounts) determined by each administration route were analyzed for statistical significance by one-way analysis of variance (ANOVA) with *p* < 0.05 indicating a significant difference. That is, *p* < 0.05 by ANOVA analysis meant that the values of the three administration routes were different. Additionally, statistical significance comparisons between each administration group were performed through multiple comparison (as post-hoc analysis; Tukey method), and *p* < 0.05 meant a significant difference. Statistical analyses of pharmacokinetic parameter values were performed for lactone and total forms of topotecan.

## 5. Conclusions

The development of new drugs and improvements to formulations are clearly important tasks, but the development and exploration of other routes of administration for existing drugs are also critical. Furthermore, the pharmacokinetic profiles obtained through other routes of administration can be of assistance in the development of new drugs as well as in formulation. Topotecan’s approved routes of administration are oral and intravenous and to date, few pharmacokinetic studies of alternative routes of administration have been reported. Considering that topotecan is actively used in the clinic for treatment of various types of cancer, our study, highlighting the pharmacokinetic and other advantages of subcutaneous administration, makes an important contribution.

According to the present study, subcutaneous administration of topotecan showed very good bioavailability (with AUC values similar to those for intravenous administration). In addition, the persistence of topotecan in blood over time after subcutaneous administration was relatively better than intravenous and oral administration. More studies will need to be performed in the future, but if there is a significant correlation between the duration (of topotecan blood concentration over time after administration) and treatment outcomes, the pharmacokinetic profiles obtained by subcutaneous administration in this study could be of enormous significance for both the clinical and formulation development fields.

## Figures and Tables

**Table 1:** Pharmacokinetic parameters of topotecan (as lactone form) and urinary and fecal excretion studies after oral, intravenous, and subcutaneous administration of 4 mg/kg topotecan hydrochloride to rats (mean ± SD, *n* = 5).

Parameters	Units	Oral	Intravenous	Subcutaneous	ANOVA (*p*-Value)
AUC_0–24_	h·ng/mL	283.71 ± 33.20	1629.02 ± 402.59	1404.53 ± 64.45	0.0000
AUC_0–∞_	h·ng/mL	308.03 ± 32.04	1633.18 ± 402.58	1438.02 ± 62.91	0.0000
F	%	18.86 ^a^	-	88.05 ^b^	-
AUC_0–24_/AUC_0–∞_ × 100	%	92.10	99.75	97.67	-
CL or CL/F	mL/h/kg	13,097.57 ± 1344.43	2565.98 ± 593.91	2785.85 ± 121.32	0.0000
C_max_	ng/mL	42.62 ± 6.95	1167.54 ± 314.80 ^c^	142.36 ± 4.95	0.0000
C_24 h_	ng/mL	2.22 ± 0.74	0.58 ± 0.23	5.60 ± 0.35	0.0000
T_1/2_	h	7.35 ± 1.72	4.91 ± 0.63	4.14 ± 0.20	0.0012
T_max_	h	1.60 ± 0.55	0.25 ± 0.00	1.35 ± 0.60	0.0015
V_d_ or V_d_/F	mL/kg	139,772.60 ± 39296.44	18,440.26 ± 5789.12	16,642.03 ± 1418.28	0.0000
Cumulative urinary excretion amounts (up to 24 h)	ng	110,206.10 ± 34,227.59	237,511.70 ± 19,040.71	343,298.40 ± 65,668.08	0.0000
Cumulative % dose in urine (up to 24 h)	%	11.02 ± 3.42	23.75 ± 1.90	34.33 ± 6.57	0.0000
Cumulative fecal excretion amounts (up to 24 h)	ng/g	14,262.94 ± 4878.87	5177.26 ± 854.26	4056.32 ± 1200.95	0.0002
Cumulative % dose in feces (up to 24 h)	%	3.30 ± 0.84	1.32 ± 0.20	1.09 ± 0.19	0.0011

^a^ Oral bioavailability (F, %) was calculated by AUC_0–∞_ of oral/AUC_0–∞_ of intravenous × 100; ^b^ Subcutaneous bioavailability (F, %) was calculated by AUC_0–∞_ of subcutaneous/AUC_0–∞_ of intravenous × 100; ^c^ C_max_ values for intravenous administration were observations at 0.25 h (as first point of blood sampling).

**Table 2:** Pharmacokinetic parameters of topotecan (as total topotecan) and urinary and fecal excretion studies after oral, intravenous, and subcutaneous administration of 4 mg/kg topotecan hydrochloride to rats (Mean ± SD, *n* = 5).

Parameters	Units	Oral	Intravenous	Subcutaneous	ANOVA (*p*-Value)
AUC_0–24_	h·ng/mL	537.87 ± 69.36	2530.59 ± 931.56	2465.78 ± 459.45	0.0003
AUC_0–∞_	h·ng/mL	599.05 ± 74.75	2540.92 ± 933.77	2534.47 ± 459.72	0.0003
F	%	23.58 ^a^	-	99.75 ^b^	-
AUC_0–24_/AUC_0–∞_ × 100	%	89.79	99.59	97.29	-
CL or CL/F	mL/h/kg	6760.67 ± 839.39	1741.12 ± 574.38	1621.16 ± 298.09	0.0000
C_max_	ng/mL	69.79 ± 16.19	1632.14 ± 586.44 ^c^	246.07 ± 27.11	0.0000
C_24 h_	ng/mL	5.39 ± 0.70	1.36 ± 0.82	10.50 ± 1.30	0.0000
T_1/2_	h	8.55 ± 2.95	5.37 ± 0.70	4.56 ± 0.55	0.0096
T_max_	h	2.00 ± 0.00	0.25 ± 0.00	1.40 ± 0.55	0.0000
V_d_ or V_d_/F	mL/kg	82,732.36 ± 26,530.66	13,820.03 ± 5621.62	10,827.60 ± 3053.30	0.0000
(C_max_ of total − C_max_ of lactone form)/C_max_ of total × 100	%	37.60 ± 10.40	26.26 ± 9.14	41.49 ± 7.60	0.0538
(AUC_0–24_ of total − AUC_0–24_ of lactone form)/AUC_0–24_ of total × 100	%	47.08 ± 3.82	33.35 ± 8.02	41.68 ± 9.45	0.0399
(AUC_0–∞_ of total − AUC_0–∞_ of lactone form)/AUC_0–∞_ of total × 100	%	48.33 ± 4.49	33.46 ± 7.99	41.98 ± 9.10	0.0263
Cumulative urinary excretion amounts (up to 24 h)	ng	130,993.70 ± 29,686.67	558,753.60 ± 88,382.01	683,097.00 ± 67,062.19	0.0000
Cumulative % dose in urine (up to 24 h)	%	13.10 ± 2.97	55.88 ± 8.84	68.31 ± 6.71	0.0000
Cumulative fecal excretion amounts (up to 24 h)	ng/g	18,068.43 ± 6171.61	6601.36 ± 1643.26	4623.71 ± 1090.77	0.0002
Cumulative % dose in feces (up to 24 h)	%	4.07 ± 0.68	1.66 ± 0.29	1.25 ± 0.20	0.0002

^a^ Oral bioavailability (F, %) was calculated by AUC_0–∞_ of oral/AUC_0–∞_ of intravenous × 100; ^b^ Subcutaneous bioavailability (F, %) was calculated by AUC_0–∞_ of subcutaneous/AUC_0–∞_ of intravenous × 100; ^c^ C_max_ values for intravenous administration were observations at 0.25 h (as first point of blood sampling).

## Supplementary Materials

Supplementary materials can be found at https://www.mdpi.com/1424-8247/13/9/231/s1. Figure S1: Chemical structures of the lactone and carboxylated forms of topotecan hydrochloride, Figure S2: Positive precursor and product ion mass spectra of topotecan lactone form (A) and repaglinide as an IS (B) in UPLC-ESI-MS/MS quantification, Figure S3: Chromatograms in rat plasma (A), urine (B), and feces (C) of blank samples (a), zero samples containing IS (b), blank samples containing LLOQ of topotecan and IS (c), the obtained samples after administration of 4 mg/kg topotecan hydrochloride (d). (A) plasma (a: blank, b: zero, c: LLOQ, d: the obtained sample at 0.5 h after intravenous administration of 4 mg/kg topotecan hydrochloride) (B) urine (a: blank, b: zero, c: LLOQ, d: the obtained sample at 6 h after subcutaneous administration of 4 mg/kg topotecan hydrochloride) (C): feces (a: blank, b: zero, c: LLOQ, d: the obtained sample at 24 h after oral administration of 4 mg/kg topotecan hydrochloride), Figure S4: Calibration curves in rat plasma (A), urine (B), and feces (C) for quantification of topotecan (*n* = 5). Vertical bars represent standard deviation of the mean, Table S1: Accuracy and precision results of topotecan quantification in intra- and inter-batch (*n* = 5), Table S2: Recovery and matrix effect results in topotecan assay (Mean ± SD, *n* = 5), Table S3: Stability test results of topotecan in rat matrices (Mean ± SD, *n* = 5).

## Abbreviations

UPLC-ESI-MS/MS	Ultraperformance liquid chromatography-electrospray ionization-tandem mass spectrometer
IS	Internal standard
ESI	Electrospray ionization
PP	Protein precipitation
AUC	Area under the curve
CL	Clearance
ANOVA	Analysis of variance
LLOQ	Lower limit of quantitation
SD	Standard deviation
MRM	Multiple reaction monitoring
SPSS	Statistical Package for the Social Sciences
