# Supplementary material for: Pharmacokinetic Comparison of Three Different Administration Routes for Topotecan Hydrochloride in Rats
Source: Pharmaceuticals (Basel). 2020 Sep 2;13(9):231. doi: 10.3390/ph13090231 (PMC7559546; doi:10.3390/ph13090231)
Supplement: Supplementary file 1 [file pharmaceuticals-13-00231-s001.pdf]

pharmaceuticals

MDPI

Supplementary Materials

Table 1. Accuracy and precision results of topotecan quantification in intra- and inter-batch (n = 5).

Matrix	Intra-batch								Inter-batch							
	Precision (CV, %)				Accuracy (%)				Precision (CV, %)				Accuracy (%)			
	LLOQ	Low QC	Medium QC	High QC	LLOQ	Low QC	Medium QC	High QC	LLOQ	Low QC	Medium QC	High QC	LLOQ	Low QC	Medium QC	High QC
Plasma	2.06	2.77	3.92	3.94	97.00	100.07	98.82	101.55	5.24	3.99	4.77	4.92	101.00	98.07	103.51	99.57
Urine	3.49	3.56	5.43	4.98	100.67	99.93	101.19	98.88	5.82	4.78	5.22	2.64	97.67	98.33	100.83	100.74
Feces	5.27	4.94	6.66	4.63	97.33	101.87	98.41	100.10	5.02	5.70	6.86	5.51	98.33	100.40	96.98	102.40

LLOQ: topotecan of 0.1 ng/mL; Low QC: topotecan of 0.3 ng/mL; Medium QC: topotecan of 500 ng/mL; High QC: topotecan of 800 ng/mL.

Table 2. Recovery and matrix effect results in topotecan assay (Mean ± SD, n = 5).

Matrix	Recovery (%)			Matrix effect (%)		
	Low QC	Medium QC	High QC	Low QC	Medium QC	High QC
Plasma	90.26 ± 1.96	91.82 ± 3.13	90.33 ± 2.41	100.32 ± 2.70	98.71 ± 3.34	99.91 ± 1.23
Urine	92.00 ± 4.06	88.90 ± 2.47	89.27 ± 4.31	96.10 ± 1.46	97.89 ± 1.93	96.87 ± 1.27
Feces	90.08 ± 3.18	90.37 ± 4.04	90.33 ± 2.55	99.03 ± 2.88	98.26 ± 3.90	100.31 ± 0.95

Low QC: topotecan of 0.3 ng/mL; Medium QC: topotecan of 500 ng/mL; High QC: topotecan of 800 ng/mL.

Table 3. Stability test results of topotecan in rat matrices (Mean ± SD, n = 5).

Matrix	Short-term stability (25°C, 24 h)		Long-term stability (-80°C, 4 weeks)		Freeze-thaw stability (-80 or 25°C, 3 cycles)		Autosampler stability (15°C, 24 h)	
	Low QC	High QC	Low QC	High QC	Low QC	High QC	Low QC	High QC
Plasma	98.97 ± 2.95	100.13 ± 1.41	96.66 ± 2.62	97.68 ± 2.12	99.21 ± 4.05	98.38 ± 2.67	100.32 ± 0.87	98.78 ± 1.35
Urine	98.03 ± 1.59	99.03 ± 3.24	98.72 ± 2.37	99.32 ± 3.05	101.05 ± 1.62	96.33 ± 5.32	98.74 ± 3.06	100.45 ± 2.09

**Feces**	99.70 ± 3.15	100.29 ± 3.46	100.69 ± 2.19	97.39 ± 1.78	99.41 ± 0.86	99.05 ± 2.37	98.21 ± 1.57	99.74 ± 2.29

Low QC: topotecan of 0.3 ng/mL; High QC: topotecan of 800 ng/mL.

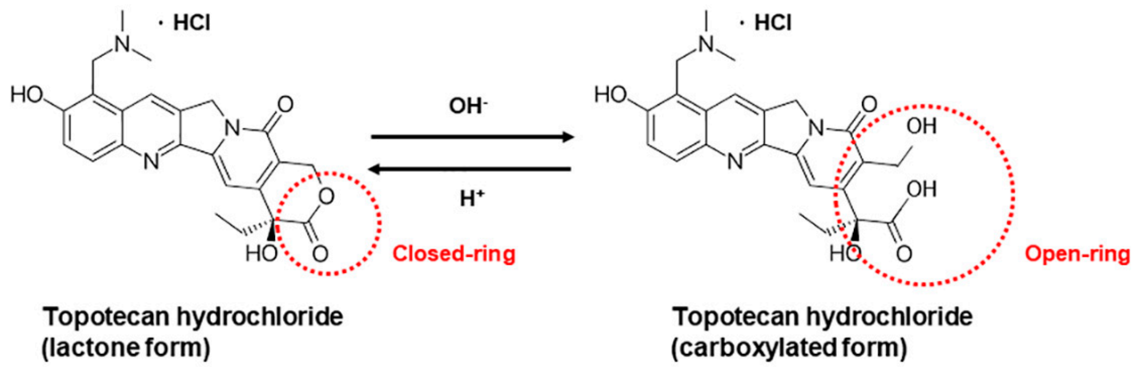

Figure 1. Chemical structures of the lactone and carboxylated forms of topotecan hydrochloride.

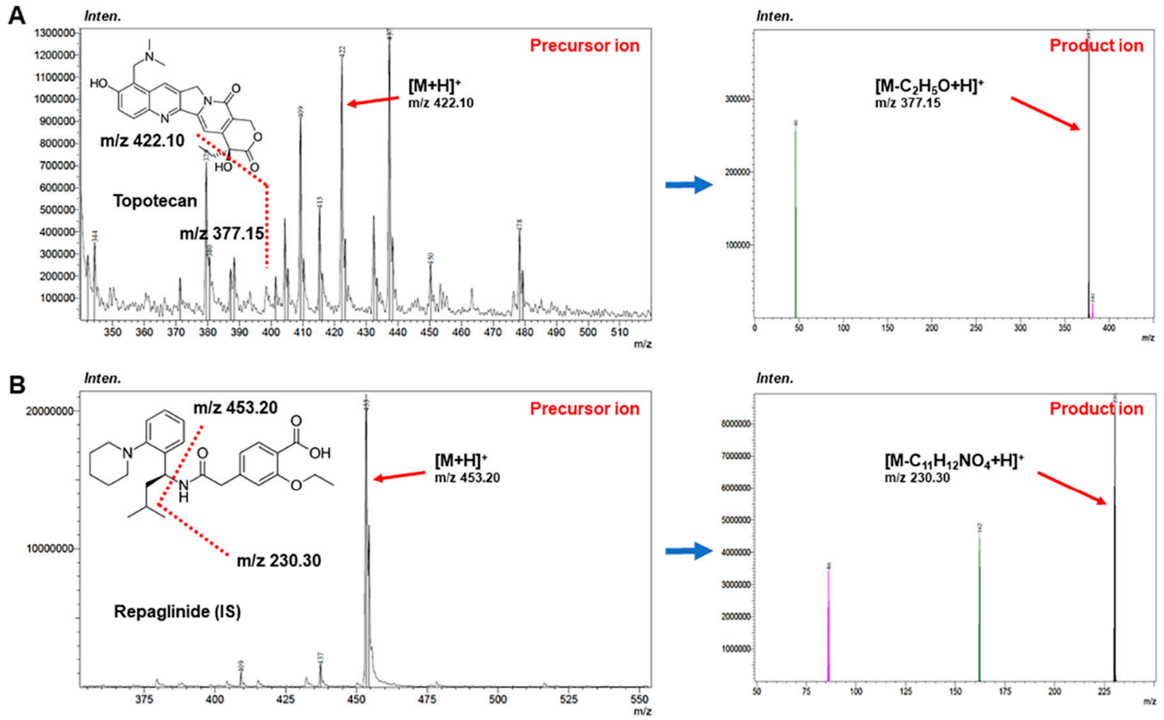

Figure 2. Positive precursor and product ion mass spectra of topotecan lactone form (A) and repaglinide as an IS (B) in UPLC-ESI-MS/MS quantification.

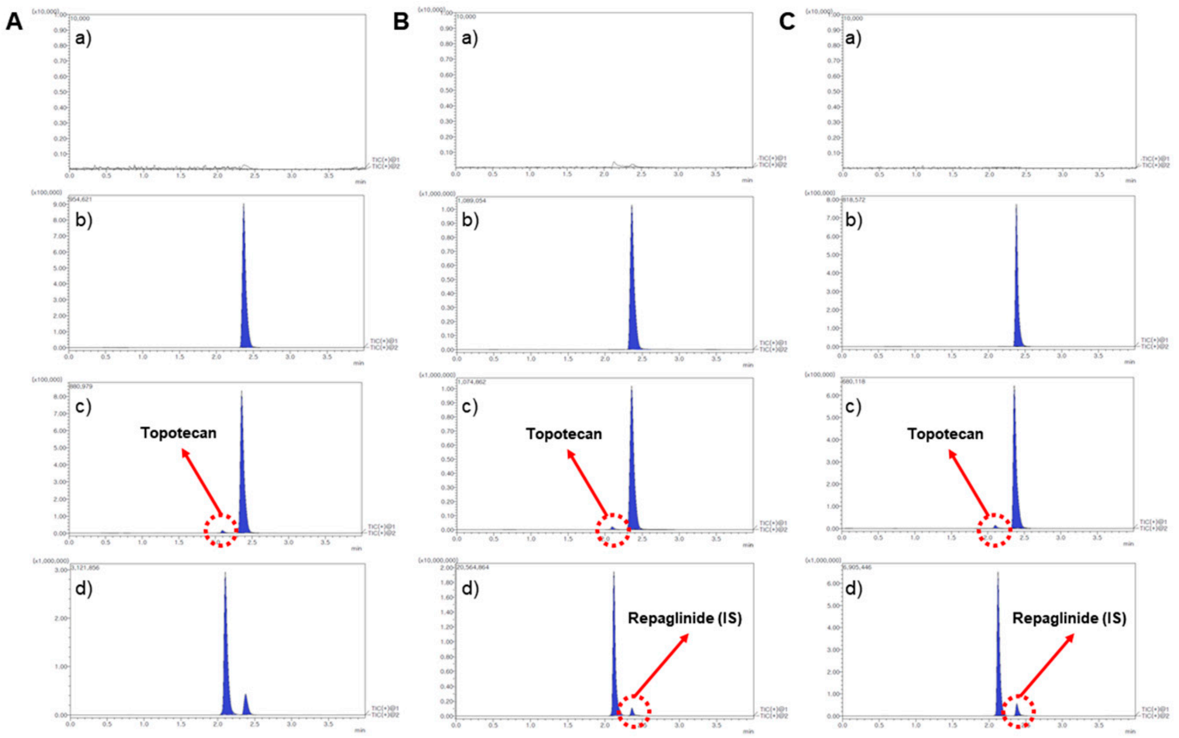

Figure 3. Chromatograms in rat plasma (A), urine (B), and feces (C) of blank samples (a), zero samples containing IS (b), blank samples containing LLOQ of topotecan and IS (c), the obtained samples after administration of 4 mg/kg topotecan hydrochloride (d). (A) plasma (a: blank, b: zero, c: LLOQ, d: the obtained sample at 0.5 h after intravenous administration of 4 mg/kg topotecan hydrochloride); (B) urine (a: blank, b: zero, c: LLOQ, d: the obtained sample at 6 h after subcutaneous administration of 4 mg/kg topotecan hydrochloride); (C) feces (a: blank, b: zero, c: LLOQ, d: the obtained sample at 24 h after oral administration of 4 mg/kg topotecan hydrochloride).

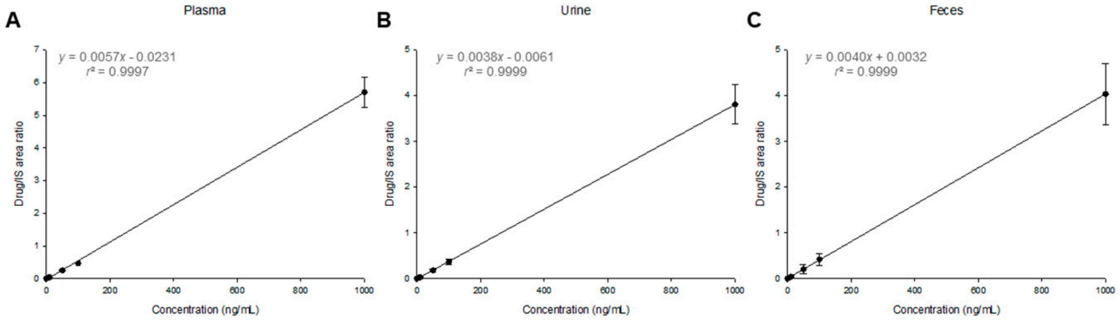

Figure 4. Calibration curves in rat plasma (A), urine (B), and feces (C) for quantification of topotecan (n = 5). Vertical bars represent standard deviation of the mean.
